# Impact of Rearing Substrates on Black Soldier Fly Growth and Fertility: A Semi-Industrial Scale Study to Optimize Egg Collection

**DOI:** 10.3390/insects16020142

**Published:** 2025-02-01

**Authors:** Qi-Hui Zhang, Nalini Puniamoorthy

**Affiliations:** Department of Biological Sciences, National University of Singapore, 16 Science Drive 4, Singapore 117558, Singapore; zqihui@u.nus.edu

**Keywords:** *Hermetia illucens*, egg production, egg hatchability, reproductive performance

## Abstract

Juvenile environments can influence adult phenotypes in holometabolous insects. This study examines the effects of larval rearing substrates on the reproductive outcomes of Black Soldier Flies (BSFs) at a semi-industrial scale. Larvae were reared on five substrates. Larval growth, adult size, and reproductive performance were monitored, with a specific focus on egg production and hatching rates across 17 continuous egg collection days. Egg production was positively correlated with female body weight, while male body weight appeared to influence the egg production peak. Extended larval feeding stages and shorter non-feeding periods were associated with an earlier onset of egg-laying, suggesting life-history traits as predictors for reproductive timing. Diets high in carbohydrates but low in protein and fat positively influenced larval growth, egg production, and female body weight, while diets rich in both protein and carbohydrates negatively affected all measured parameters. Notably, higher fecundity did not necessarily correlate with fertility (i.e., more viable larvae for bioconversion), highlighting the need to optimize diets for both egg quantity and hatchability to maximize productivity. These findings provide valuable insights for optimizing larval nutrition and egg collection practices, enhancing reproductive efficiency and overall productivity in industrial BSF farming.

## Simple Summary

Black Soldier Flies (BSF) are widely used for sustainable waste management and the production of nutrient-rich resources like animal feed. While much research has focused on how different diets affect BSF larvae, less is known about how these diets influence adult reproduction, a critical factor for farming efficiency. This study examines how larval diets affect growth, egg production, and hatching rates over a 17-day collection period in a semi-industrial setting. We found that diets rich in carbohydrates but low in protein and fat improved larval growth, female body weight, and egg production, while diets high in both protein and carbohydrates negatively impacted these parameters. Larger female flies produced more eggs, but higher egg production did not always translate to better hatchability. The timing of egg-laying was influenced by larval feeding duration and development stages. These findings provide valuable insights for optimizing BSF farming practices by identifying balanced diets that enhance both egg production and hatchability, improving waste recycling and industrial larval production efficiency.

## 1. Introduction

The black soldier fly (BSF, *Hermetia illucens*) has gained attention as an effective agent for bio-waste management and the production of nutrient-rich agricultural inputs [1]. BSF larvae are known for their ability to efficiently reduce organic waste, with reductions ranging from 50% to 80% within 10 to 14 days, depending on the waste type [2]. Additionally, the larvae’s nutrient profile is impressive, containing 35% to 57% protein and 15% to 49% lipid [3,4], making them a valuable resource for animal feed and other bio-based products [5]. The remnants of the BSF bioconversion, frass, can also be used as fertilizers in agri/horticulture. These characteristics, combined with the benign nature of BSF adults—which do not require feeding and pose minimal risk as disease vectors [6]—have firmly solidified BSF as a cornerstone of the insects-as-feed industry.

Two critical stages of the BSF life cycle hold particular importance in industrial farming: the larval stage and the adult stage [7]. The larval stage determines the efficiency of bio-waste conversion into larval biomass [8], directly affecting the productivity of BSF farming systems. The adult stage, on the other hand, governs the reproductive success of the colony, influencing factors such as egg production and egg-hatching rates [9]. Numerous laboratory and industrial-scale studies have focused on understanding the effects of rearing substrates on larval growth and development. These studies have tested a wide range of substrates, including organic kitchen waste [10,11], fish waste [12,13,14], manure [11,15], spent grains [16,17], and agricultural by-products [18,19], each with varying impacts on larval performance and nutrients.

However, the adult stage of BSF remains less understood despite its crucial role in determining reproductive success. As outlined by reviews [7,9], adult BSF reproduction is complex, and fertility is often highly variable with females even laying clutches that do not hatch [20]. Such variability can pose a challenge for mass-rearing operations that rely on a steady supply of fertile eggs for neonate larvae production. Thus, understanding how environmental factors, such as larval-rearing substrates affect adult reproductive success is crucial for scaling up BSF production to meet the growing demand for larvae in the feed industry.

To date, research on the effects of larval substrates on the adult stage, particularly in terms of adult reproductive performance, remains limited to a few laboratory-scale studies. For instance, Singh et al. [21] examined the impact of rearing substrates on adult and egg development time using 400 larvae per replicate. An earlier study examined the effect of substrates on individual female egg production using a single egg collection event [22]. Laursen et al. [23] conducted a full-scale industrial rearing under various larval diets, using subsets of 200 adults per mating cage, with egg production data collected on four specific dates instead of continuously. While these studies demonstrated that larval substrates influence adult-stage egg production and development, they do not adequately capture the complexities and dynamics of BSF reproduction at an industrial scale.

In industrial BSF rearing, the production scale is far greater than in lab-scale tests and eggs are typically collected over multiple days rather than from a single egg collection event. To the best of our knowledge, no studies have yet bridged this lab–industry gap on how the rearing substrates influence egg hatching, a critical factor that determines overall reproductive success. In this study, we evaluated the effects of five different substrates on both larval growth and adult reproduction at a semi-industrial scale. Obtained results offer valuable insights into how different rearing substrates influence reproductive outcomes in BSF, providing knowledge for optimizing industrial egg collection practices and enhancing overall colony maintenance efficiency.

## 2. Materials and Methods

### 2.1. Experimental Materials and Location

The experiments were conducted at a facility of the biotechnology company Ento Industries Pte. Ltd., located in Singapore (1°17′04.700″ N, 103°49′12.400″ E). The eggs of BSF were provided by the same company and the handling of the neonates after hatching followed usual industry practices, as the larvae were reared for five days on a standard nursery diet (commercial chicken feed). After the five-day period, larvae were transferred to the different experimental substrates.

Five experimental meals with distinct nutrient profiles were prepared (Table 1). The macronutrient content, including protein, carbohydrates, and lipids, was analysed following the methods described in this study [10].

### 2.2. Experimental Setup

To evaluate the correlation between larval number and weight, 5-day-old neonates were measured in 10 replicates, with approximately 300–600 individuals per replicate. A total of 1500 5-day-old larvae were weighed and transferred to individual rearing containers (30 × 40 × 10 cm), resulting in a larval density of 0.8 cm^2^ per larva. Each container held a single feeding of 1500 g (1 g per larva) of the experimental meal containing 70–80% relative moisture to ensure a sufficient feed supply for the larvae. Each treatment had three replicates. All experiments were conducted at room temperature, 30–32 °C, with an environmental humidity of 69–71% relative humidity (%RH).

### 2.3. Sampling and Analysing

Twenty larvae were randomly selected every two days from each container, gently washed with tap water, and dried using kitchen towels. The mass of selected larvae was recorded with a precision balance (0.001 g accuracy, PR312E, Lab1st Scientific, Irvine, CA, USA). After weighting, the larvae were returned to their respective container. The duration of the larval stage was measured as the number of days from the start of the experiment to the first appearance of prepupae, a late-stage larva characterised by the cessation of feeding and a distinctive dark brown colouration [7]. The non-feeding duration, including the period from prepupal appearance to adult emergence, was recorded from the day prepupae were first observed until adults emerged. Once the larvae reached the prepupal stage, all prepupae/pupae from each replicate were transferred to a mating cage. Sixty-five pupae from each replicate were randomly collected and placed individually in small containers. Adult body weight was recorded immediately after emergence, after which the flies were returned to the mating cages.

**Figure 1:**
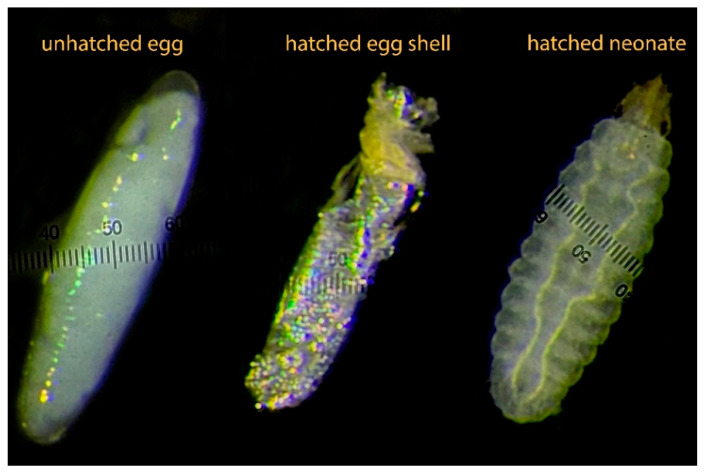
Image showing an unhatched egg, a hatched empty eggshell, and a newly hatched black soldier fly (BSF) neonate.

Once adults began emerging, the mating cages were placed under natural light to facilitate mating, and the cages were rotated daily to minimize location effects. Egg traps were positioned above the chicken feed, used as an attractant, and placed inside each mating cage. The traps were collected and replaced daily to measure the egg production for each treatment. Randomly selected egg clutches were incubated for four days to allow hatching, after which they were frozen. Egg hatching rates were measured under a microscope (Figure 1), and a subsample of eggs was weighed to determine individual egg weight.

### 2.4. Statistical Analysis

**Figure 2:**
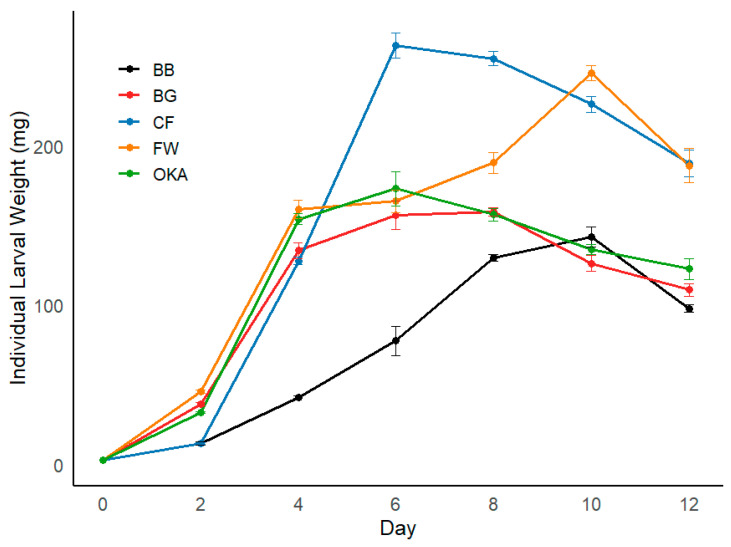
Growth curves showing individual larval weight changes across different meal treatments. Each treatment is represented by a different colour. Error bars represent standard errors.

Data were analysed using ANOVA to compare means among larval meal treatments, followed by Tukey’s HSD test for post-hoc comparisons. Principal component analysis (PCA) was conducted to identify the main patterns in the nutrient profiles of the diets. Regression analysis was then performed to investigate the effects of PC1 and PC2 on prepupal weight, accumulated egg weight at day five and day ten, and adult body weight, with model fit assessed using adjusted *R*^2^ and AIC. All the tests and modelling were performed with R version 2024.09.0 software for Windows. All figures were generated using the ggplot2 package in the R software (4.3.1) package [24]. The larvae on BB (bread and biscuit) exhibited a very slow growth rate (details presented in Figure 2) and failed to reach the adult stage. Thus, this treatment was excluded from the downstream analyses for adult-stage sampling.

## 3. Results

### 3.1. Growth and Development for BSF Larvae and Adults in Different Substrates

Larvae reared on CF and FW diets developed into the largest prepupae, while those fed on OKA and BG produced smaller prepupae (Figure 2, Table 2). Contrarily, the larval duration did not show a consistent relationship with prepupal size. Despite being at opposite ends of the size spectrum, both CF larvae, which produced the largest prepupae, and OKA larvae, which produced the smallest, reached the prepupal stage the fastest (8 days). In contrast, the FW group, which also produced large prepupae, took the longest to reach the prepupal stage (10 days). The BG group, which produced the smallest prepupae, required an extended duration as well (9 days). The non-feeding duration (from prepupal appearance to adult emergence) was shorter for the FW and BG groups—despite their longer larval durations—compared to the CF and OKA groups, which reached the prepupal stage more quickly (Table 2). The larvae reared on the BB diet demonstrated significantly slower growth rates (Figure 2) and failed to develop into viable prepupae or pupae. Instead, they remained soft and dark yellow before succumbing, unable to progress to the adult stage.

**Figure 3:**
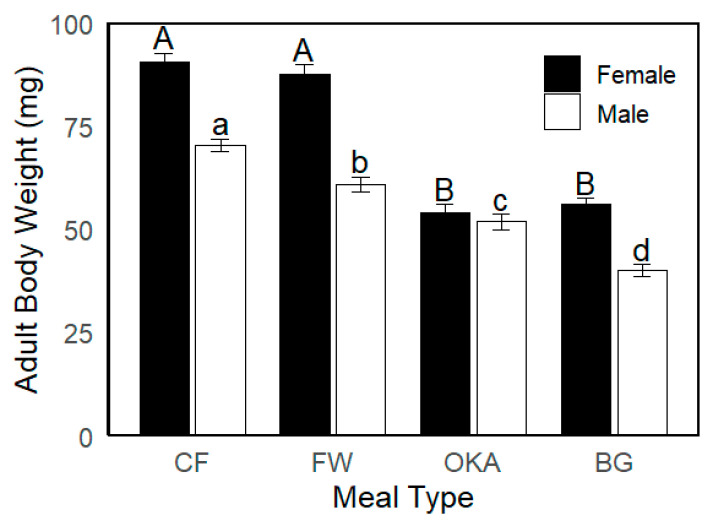
Adult body weight of black soldier fly (BSF) reared on different larval meal treatments, with sexes differentiated by bar colour. Uppercase letters indicate significantly different means for females, and lowercase letters indicate significantly different means for males (Tukey’s test, *p* < 0.05). Error bars represent standard errors.

Larger larvae and prepupae led to larger female adults. Larvae reared on CF and FW substrates developed into the largest female adults, while those fed on OKA and BG produced smaller females (Figure 3). Male body weight, however, showed more variation across the different larval meal treatments (Figure 3). The heaviest larvae from the CF group developed into the heaviest males, whereas FW larvae, despite being equally heavy as CF larvae, produced smaller adult males compared to the CF group. Although there was no significant difference in prepupal weight between the OKA and BG groups, males from the OKA group were heavier than those from the BG group. In addition, the OKA group showed the smallest difference in body weight between females and males compared to the other treatment groups (Figure 3).

### 3.2. PCA and Regression Analysis of the Impact of Diet Nutrient Profiles

Principal component analysis (PCA) identified two components explaining 100% of the variance in nutrient profiles. PC1 (60.97%) was negatively associated with protein (−0.5943) and fat (−0.7367) and positively associated with carbohydrates (0.3226), representing diets rich in carbohydrates but low in protein and fat. PC2 (39.03%) was positively associated with both protein (0.5497) and carbohydrates (0.8315), with minimal contribution from fat (−0.0794), representing diets rich in protein and carbohydrates.

The regression results showed that including both PC1 and PC2 in the model provided a better fit, as indicated by a lower AIC and higher adjusted *R*^2^ (Table 3). PC1 was positively (Coefficient > 0) associated with prepupal weight, total collected egg weight by day ten, and female body weight, indicating that diets rich in carbohydrates but low in protein and fat increased these parameters. In contrast, PC2 was negatively (Coefficient < 0) associated with prepupal weight, total collected egg weight by day five and day ten, female body weight and male body weight, indicating that diets rich in both protein and carbohydrates reduced these parameters (Table 3).

### 3.3. Egg Production and Egg Hatching Rate

**Figure 4:**
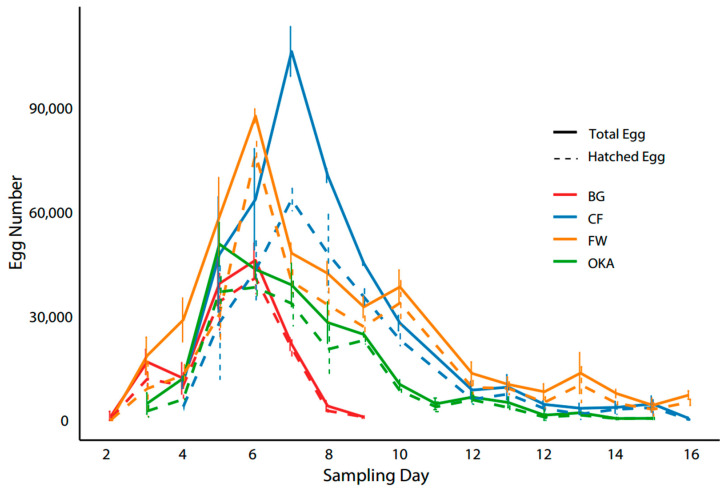
Total egg number and hatched egg number recorded on different sampling days. Solid lines represent the total egg number, while dashed lines represent the hatched egg number. Different larval meal treatment groups are indicated by colour.

Different larval diets led to distinct patterns in egg production and hatching rates across all groups, causing variations in the timing and duration of egg-laying, total egg weight, and the efficiency of egg hatching (Figure 4 and Table 2).

Total egg production accumulated by sampling Day 5 and Day 10 followed the trend seen in female adult body weight. The CF and FW groups, which had the largest females, exhibited higher egg production, while the OKA and BG groups, with smaller females, produced significantly fewer eggs (Figure 3 and Table 2). By Day 5, the egg weight for the CF and FW groups was twice that of the BG group, and by Day 10 it was three times higher than the BG group’s egg production (Table 2).

FW and BG, which had longer larval durations and shorter non-feeding durations, were the first groups to start laying eggs, both achieving peak production within five days (Figure 4). However, while the total egg production period for the BG group (with the smallest larvae and adults) lasted only eight days, the FW group (with the largest larvae and females) extended beyond 16 days (Figure 4). OKA and CF began egg-laying later than the FW and BG groups but reached peak egg production more quickly. OKA achieved its peak within two days and sustained production for 15 days, though egg output declined significantly after Day 8. CF reached its peak egg production within three days and sustained production over 16 days (Figure 4).

In the CF, FW, and BG groups, the peak in hatched egg numbers coincided with the peak in total egg production. However, in the OKA group, the peak in hatched eggs occurred one day after the egg production peak. The CF group experienced the lowest egg-hatching rate at the time of their peak egg production, with the gap between total egg numbers and hatched eggs gradually narrowing in the following days. In the FW group, egg hatching rates were initially very low before the egg production peak, but from that point onward—including on the peak day—the gap between hatched and total eggs remained small. OKA adults, in contrast, the hatching rate dropped sharply at the egg production peak but later stabilised at a high level, especially from Day 8 onward. Notably, while CF produced more eggs at its peak (SD 6) compared to FW’s peak (SD 5), FW had a higher number of hatchable eggs than CF (Figure 4).

## 4. Discussion

Variations in larval stage nutrition resulted in differing male and female body weight responses, suggesting a potential energy allocation trade-off. In the CF group, both males and females were the heaviest among all treatments. In the FW group, females were comparable in size to those in the CF group, but the males were smaller. Similarly, in the BG group, females were similar in size to those in the OKA group, but BG males were smaller than OKA males. In BSFs, resource allocation tends to be female-biased, with females typically being heavier than males [25]. Our results suggest that when larval nutrition varies, this bias may become more pronounced, with limited resources preferentially allocated to females. This is likely due to the high costs associated with egg production, which directly contributes to reproductive success [26,27].

This potential fitness advantage of female-biased resource allocation is further supported by our egg production data, as heavier females exhibited higher total egg output. This suggests that larger female body size, resulting from more optimal larval diets, directly enhances reproductive success. Supporting this, previous studies have demonstrated a positive correlation between high-energy diets and increased adult body weight, which leads to larger egg clutches [26,28]. This is likely due to larger BSF females possessing larger ovaries, thereby increasing their reproductive capacity [22,29]. Our results further underscore the need for optimised larval nutrition strategies to enhance the reproductive efficiency of BSF colonies in industrial farming.

High dietary protein has been reported to negatively affect adult emergence [30]. In our study, a high-lipid diet (BB diet) was also found to prevent larvae from pupating and emerging as adults, likely due to excess fat creating anaerobic, sticky conditions that impaired larval respiration and growth [31]. The PCA and regression analyses further reveal how diet nutrient profiles influenced larval and adult-stage outcomes. Diets rich in carbohydrates but low in protein and fat resulted in larger prepupal weight, higher egg production, and greater female body weight, suggesting that carbohydrate-dominant diets support both growth and reproduction. In contrast, diets with higher levels of both protein and carbohydrates were associated with reduced prepupal weight, lower egg weight by day five and ten, and smaller adult body weight in both males and females. Previous studies reported that higher protein content increased BSF egg yield but also highlighted the role of the protein-to-carbohydrate ratio in influencing reproduction and adult lifespan [32,33]. Further studies are needed to explore the optimal dietary nutrient profiles to enhance both larval growth and reproductive outcomes in BSF farming.

FW and BG groups, which experienced extended larval stages coupled with shorter non-feeding periods, began laying eggs earlier. In contrast, CF and OKA, with shorter larval durations and prolonged non-feeding periods, delayed the start of egg-laying. The results reveal a relationship between early-stage development and reproductive timing: longer larval durations (feeding stage) were associated with shorter non-feeding durations (prepupal and pupal stages), resulting in an earlier onset of egg-laying. Previous research has demonstrated that BSF larval and pupal durations vary depending on the larval stage diets [23,34]. Yet, the correlation between larval diet nutrition and life-history traits is usually complex. Suitable larval diets can extend the feeding stage, enabling larvae to accumulate greater reserves and reach larger sizes for pupation [10], whereas the absence of critical growth factors in the diet may lead to reduced pupal weight and prolonged larval development [23,35]. Consequently, relying on diet nutrient profiles alone to predict egg production timing poses challenges for industrial applications. This study is the first to link BSF life-history traits, such as larval and non-feeding durations, with population-level egg production timing. Practically, the emergence of prepupae could serve as a potential indicator for predicting egg production in industrial-scale BSF farming.

Despite both OKA and CF showing a delay in the start of egg-laying, the smaller OKA group reached peak egg production sooner than the CF group, which had the largest female and male adults. A previous study found that smaller males can have shorter mating latency and higher mounting accuracy [22], which may explain why smaller OKA flies reached peak production sooner. Smaller males might lead to quicker mating success, which in turn facilitates faster egg maturation and earlier egg-laying by females [36,37]. Female conditions could also be a key driver of reproductive timing. In BSF, females are polyandrous [38], and larger females that can carry more eggs [29], thus may require more matings to ensure fertilization. This increased demand for mating might account for the delayed egg-laying and egg-production peak observed in the CF group. Future studies could investigate how larval stage nutrition influences the degree of polyandry in BSFs and its impact on female fecundity.

Notably, higher egg production does not necessarily translate to a greater number of viable larvae available for bioconversion. In our study, we found that although the CF group produced more eggs compared to the FW group at their peaks, the FW group yielded a higher number of hatchable eggs. This discrepancy highlights that egg viability and hatchability are crucial factors in determining the overall efficiency and success of BSF farming operations, beyond just the quantity of eggs produced. BSF industrial practices should prioritize substrates that enhance both egg output and hatchability through targeted dietary adjustments, maximizing overall larvae productivity for bioconversion.

Larval diets that produced heavier females and higher initial egg production tended to have lower egg hatching rates early on, with the hatching rate gradually increasing over time. In contrast, diets that resulted in smaller females showed a higher hatching rate from the start, but their overall egg production period was shorter. This indicates that in industrial settings, the timing of egg collection is crucial, as both the total egg production and the number of hatched neonates can vary significantly depending on the day of collection and are further influenced by the larval diet. Optimizing the timing and frequency of egg collection, based on the specific diet provided to larvae, can therefore have a major impact on production efficiency.

This is the first study to provide a detailed examination of how different larval diets affect both male and female body weight and reproductive outcomes in BSFs at a semi-industrial scale. The insights gained here can guide egg collection practices in BSF farming, ensuring optimal timing to maximize egg yield and hatchability for enhanced operational efficiency.

## 5. Conclusions

This study highlights the significant impact of larval diets on both female and male body weights, and how these differences influence reproductive outcomes in BSF farming. Larger female body weight, resulting from more suitable larval diets, was significantly correlated with higher egg production. Yet, our results also indicate that higher egg production does not guarantee more viable larvae for bioconversion. Thus, in industrial BSF farming, optimizing rearing substrates to enhance both egg output and hatchability is crucial for maximizing overall productivity.

Our findings also suggest that BSF life-history traits, including larval and non-feeding durations, may serve as predictors for the onset of egg-laying, with extended larval feeding stages and shorter non-feeding periods associated with an earlier start to egg-laying. Additionally, male body weight appears to influence the timing of peak egg production, with smaller males associated with an earlier peak and higher initial hatching rates. Potential caveats include the challenge of tracking sex ratios as well as individual mortality in high-density cages. Future studies could explore the influence of operational sex ratios on female fecundity and longevity in an industrial setting. Understanding these dynamics provides valuable insights for refining egg collection practices, enabling the optimisation of both the quantity and timing of egg production for enhanced efficiency in industrial BSF farming.

## Figures and Tables

**Table 1:** Macronutrient composition, formulation, and material source of different larval meals.

Meal	Formulation	Source of Materials	Moisture (% of DM)	Crude Protein (% of DM)	Crude Carbohydrate (% of DM)	Crude Fat (% of DM)
CF	chicken feed (100%)	commercial purchase	70%	19.56	55.53	6.07
HF	hall food waste (100%)	heterogeneous hawker centre waste	75%	16.63	59.28	14.67
OKA	okara (100%)	soybean processing waste	76%	27.06	55.14	9.41
BG	brewed Grains (100%)	brewing waste	77%	24.97	60.56	6.26
BB	bread (50%) and biscuits (50%)	leftover bakery products	70%	12.66	33.65	27.02

Note: The formulation of the meals represents the percentage of dry material content before mixing with water.

**Table 2:** Effects of different larval meal treatments on prepupal weight, development time, and egg production.

Meal	Prepupal Weight	1st pp (Day)	1st pp to 1st Adult (Day)	Egg Weight_D5 (g)	Egg Weight_D10 (g)
CF	148.16 ± 3.31 a	8	12	7.64 ± 1.84 a	10.10 ± 1.63 a
FW	140.11 ± 2.37 a	10	9	6.18 ± 0.78 ab	9.69 ± 0.91 a
OKA	98.84 ± 9.49 b	8	11	4.43 ± 0.65 b	5.76 ± 0.67 b
BG	83.21 ± 5.54 b	9	10	3.49 ± 0.17 b	3.62 ± 0.19 b

Note: 1st pp represents the duration (in days) from the start of the experiment to the first emergence of prepupae, serving as an indicator of larval duration. 1st pp to 1st adult represents the duration (in days) from the first prepupa to the first adult emergence, serving as an indicator of the non-feeding duration. Egg weight_D5 and Egg weight_D10 represent the total egg production (g) from the onset of egg production to days five and ten, respectively. For prepupal weight, Egg weight_D5, and Egg weight_D10 values are expressed as mean ± standard error and letters indicate significant differences based on the Tukey HSD test.

**Table 3:** Summary of PCA Regression Results for Nutrient Influence on Measured Parameters.

Variable	Model	Adjusted *R*^2^	AIC	PC1 Coefficient (*p*-Value)	PC2 Coefficient (*p*-Value)
Prepupal weight	PC1	0.0465	−34.32	0.0090 (0.277)	-
	PC1 + PC2	0.9509	−60.41	0.0090 (0.002)	−0.0247 (2.73 × 10^−5^)
Egg weight_D5	PC1	0.0207	42.66	0.5945 (0.315)	-
	PC1 + PC2	0.715	32.17	0.5945 (0.092)	−1.5749 (0.005)
Egg weight_D10	PC1	−0.0332	49.31	0.6857 (0.417)	-
	PC1 + PC2	0.8827	30.34	0.6857 (0.043)	−2.4989 (0.0003)
Female body weight	PC1	0.3174	78.1	8.555 (0.066)	-
	PC1 + PC2	0.929	58.34	8.555 (0.0005)	−12.426 (0.0002)
Male body weight	PC1	−0.072	77.41	2.578 (0.518)	-
	PC1 + PC2	0.859	59.77	2.578 (0.110)	−11.807 (0.0005)

Notes: Adjusted *R*^2^ indicates the proportion of variance explained by the model, with higher values reflecting a better fit. AIC measures model quality, with lower values indicating a better fit. PC1 represents diets rich in carbohydrates but low in protein and fat, while PC2 represents diets rich in both protein and carbohydrates. Positive or negative coefficients show the direction of the relationship, and *p*-values indicate statistical significance (*p* < 0.05).

## Data Availability

All relevant data and the code used for analysis in this study are available at the following repository: https://github.com/ReproLab/Data-and-codes_BSF-reproduction (accessed on 20 January 2025).
